# Mobile-Health Applications for the Efficient Delivery of Health Care Facility to People with Dementia (PwD) and Support to Their Carers: A Survey

**DOI:** 10.1155/2019/7151475

**Published:** 2019-03-27

**Authors:** Kanwal Yousaf, Zahid Mehmood, Tanzila Saba, Amjad Rehman, Asmaa Mahdi Munshi, Riad Alharbey, Muhammad Rashid

**Affiliations:** ^1^Department of Software Engineering, University of Engineering and Technology, Taxila 47050, Pakistan; ^2^Department of Computer Engineering, University of Engineering and Technology, Taxila 47050, Pakistan; ^3^College of Computer and Information Sciences, Prince Sultan University, Riyadh 11586, Saudi Arabia; ^4^College of Business Administration, Al-Yamamah University, Riyadh 11512, Saudi Arabia; ^5^College of Computer Science and Engineering, University of Jeddah, Jeddah 21577, Saudi Arabia; ^6^Department of Computer Engineering, Umm Al-Qura University, Makkah 21421, Saudi Arabia

## Abstract

Dementia directly influences the quality of life of a person suffering from this chronic illness. The caregivers or carers of dementia people provide critical support to them but are subject to negative health outcomes because of burden and stress. The intervention of mobile health (mHealth) has become a fast-growing assistive technology (AT) in therapeutic treatment of individuals with chronic illness. The purpose of this comprehensive study is to identify, appraise, and synthesize the existing evidence on the use of mHealth applications (apps) as a healthcare resource for people with dementia and their caregivers. A review of both peer-reviewed and full-text literature was undertaken across five (05) electronic databases for checking the articles published during the last five years (between 2014 and 2018). Out of 6195 searches yielded articles, 17 were quantified according to inclusion and exclusion criteria. The included studies distinguish between five categories, viz., (1) cognitive training and daily living, (2) screening, (3) health and safety monitoring, (4) leisure and socialization, and (5) navigation. Furthermore, two most popular commercial app stores, i.e., Google Play Store and Apple App Store, were searched for finding mHealth based dementia apps for PwD and their caregivers. Initial search generated 356 apps with thirty-five (35) meeting the defined inclusion and exclusion criteria. After shortlisting of mobile applications, it is observed that these existing apps generally addressed different dementia specific aspects overlying with the identified categories in research articles. The comprehensive study concluded that mobile health apps appear as feasible AT intervention for PwD and their carers irrespective of limited available research, but these apps have potential to provide different resources and strategies to help this community.

## 1. Introduction

**Figure 1:**
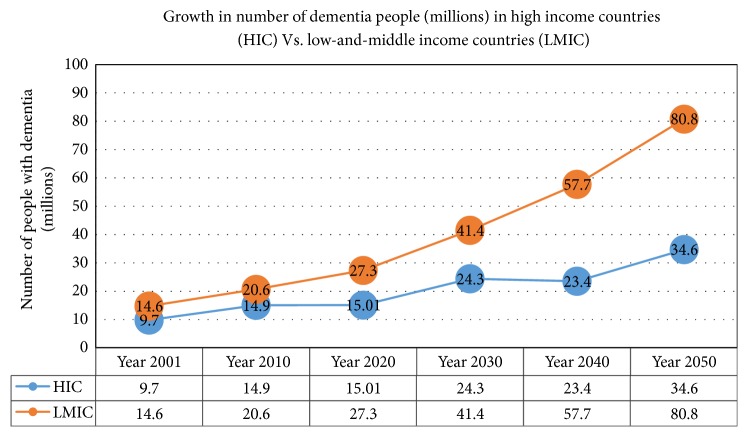
The number of people with dementia disease (millions) in high-income countries vs. low-and-middle-income countries from the year 2001 to the year 2050 [3, 4].

Dementia is considered as one of the most challenging conditions in older people that affects not only the people with this chronic illness but also their nonprofessional or informal caregivers. It is a complex syndrome with progressive decline in cognitive functioning such as thinking, remembering, and reasoning [1]. National Institute on Aging (NIA) stated that dementia is a brain disorder that disturbs the cognitive and behavioural abilities to such an extent that it hinders a person's daily living activities [2]. A study [3] based on the Delphi consensus technique estimated the global prevalence of dementia. This report stated that approximately 24.3 million people suffered from dementia in the year 2001 and this number is expected to double in every 20 years, i.e., 42.3 million PwD in 2020 and 81.1 million in 2040. Prince et al. [4] also projected the risks of rise in dementia cases from 65.7 million to 115.4 million in the duration of 2030 to 2050. The current and future estimated ratio of PwD in developing countries (low-and-middle-income countries (LMIC)) is greater than in developed countries (high-income countries (HIC)). Approximately 58% of the total PwD cases in 2010 were from developing countries (LMIC) and were estimated to upsurge to 70% in 2050. The total number of dementia people (in millions) in HIC vs. LMIC (from 2001 to 2050) is exemplified in Figure 1 by using the statistics of Ferri et al. [3] and Prince et al. [4].

Although many health organizations approved different kinds of medications for the treatment of mild to moderate dementia disease, still these medicines do not cure, reverse, or tackle the underlying root problem causing dementia. Additionally, dementia treatment consumes a large number of resources and money [5]. The treatment cost increases with the severity of dementia [6]. Moreover, PwD need care as per their severity level and the majority of which is provided by their family members who are usually inexpert for this demanding role. Family members are commonly considered as the main source of providing physical, emotional, social, and financial support, due to which they encounter different challenges like anxiety, depression, hopelessness, and poor quality of life [7]. Therefore, in the absence of a cure and limited care skills of caregivers, more advanced strategies need to be developed to maximize the quality of life and promote the independence of high need and high-cost dementia patients [8].

In this context, the assistance of technology offers much potential and can improve the quality of life of people with dementia and their informal caregivers [9]. Recent initiatives in smart devices (i.e., smartphones, portable workstations, tablets, etc.) have made mobile applications a promising source for engaging people in healthcare [10], particularly PwD with high healthcare needs [11, 12]. Mobile Health, aka mHealth, is the provision of a healthcare facility to people by using a mobile device. Approximately over 50,000 medicine related applications are available for mobile devices and the majority of these applications are free [13]. Researches [14–20] indicated that PwD can use touchscreen devices easily, and this technology is able to provide a wide range of benefits to them and their caregivers. It makes a valuable opportunity for developers to deliver a meaningful app by adding engaging activities for such people to live their life more independently.

The aim of this paper is to provide a comprehensive review of existing research that supports the evidence of using mHealth applications in dementia healthcare. The research paper is organized as follows: next section provides the background research on dementia and the role of assistive technology in dementia healthcare. Then, the underlying methodology of the research is presented and followed by the existing research studies about mHealth apps used for dementia healthcare. Afterwards, currently available smartphone apps in Android and iOS markets were listed. The paper closes with the conclusion.

## 2. Background and Related Work

Familiarity with dementia history is essential for health professionals, informal caregivers, and health policy makers. It is also important because dementia cases are elevating significantly with every passing year and intensifying the use of national resources. Roberts and Caird [21] made a correlation of Crichton Geriatric behavioural rating scale [22] with their own dementia rating scale and divided subjects into four major categories as shown in Table 1. Later on, the global numeric scale was devised [23] to quantify the severity of dementia symptoms and was termed as a clinical dementia rating (CDR) system. The cognitive and functional performance of patient screened in six areas, such as memory (M), orientation (O), judgment and problem solving (JPS), community affairs (CA), home and hobbies (HH), and personal care (PC). At the end of the screening, patients were assigned with a rating ranging from 0 (normal) to 3 (severe dementia).

As far as the connection of cost-dementia severity is concerned, Jönsson and Wimo [24] estimated that the cost associated with the care of dementia patient escalates with the severity of the disease. The findings of world Alzheimer report 2015 [25] also mentioned an increment in global cost of dementia by 35% in duration of 2010 to 2015 due to increasing dementia population and the cost of per person treatment. It is observed that carers such as family members play a pivotal role in providing the caregiving facility to PwD. This chronic disease directly influences the quality of life of a person, his/her family, and caregivers. The ratio of dementia people living in homes is higher as compared to hospitals or healthcare centres [5]. The patients living at home require extra care and monitoring [26] due to which the quality of life and psychological health of caregivers is compromised a lot [27]. To overcome this challenge, it is essential to reduce the need for the physical presence of a caregiver and still providing constant monitoring.

No doubt technology can assist in healthcare [28–32], but choosing the appropriate AT in dementia healthcare is challenging and the nature of dementia can also make a person with dementia or carer cautious or suspicious of using newer devices. In this regard, McCreadie and Tinker [33] surveyed older people (aged 70 years or above) with mild to severe mental impairments for witnessing their experience and acceptability to a wide range of ATs. Results showed the endorsement of participants towards the contribution of assistive technologies in assisting them with daily life activities and this AT promotes independence among many older people with impairments. Similarly, smart technology, i.e., smartphones and/or tablets, can also act as a potential alternative for providing healthcare solutions to dementia patients and caretakers, also known as mHealth [34]. Studies [35, 36] affirmed that smartphones or tablets are the potential solutions to meet the demands of PwD by providing simple interactive features such as a touchscreen, motion detection using motion sensors, and voice recognition. It also gives them feelings of relaxation and recreation. The qualitative exploratory study [37] was performed to find the support of touchscreen devices (e.g., phones and tablets) for people with dementia. During the sessions, it was observed that PwD have the capability of learning new skills via several coaching interventions and have proficiency in comprehending new technologies. It is also expected that self-management abilities among dementia sufferers can increase by using such apps.

Many mobile applications are incorporating the strategies defined by Alzheimer disease international (ADI) for improving healthcare facility of dementia people; the strategies are as follows [38]:

1. *Task Sharing*: transfer of care services from specialists to nonspecialists including family members
2. *Case Management*: professionals responsible for coordinating and facilitating dementia people and their carers lives
3. *Care Pathways*: systemized planning, resourcing, and delivery of care

It is identified that the provision of appropriate technology support can provide independence and improve the quality of life of elderly people with dementia disease [39].

## 3. Research Methodology

### 3.1. Research Questions

The comprehensive review aims to identify, appraise, and synthesize the existing evidence on the use of mHealth apps as dementia healthcare solution for PwD and their carers by answering the two following questions:

1. What types of mHealth apps are available for people with dementia and how do these apps help them in daily living?
2. What is the contribution of mHealth apps in supporting informal carers of people with dementia?

### 3.2. Study Design

This study is followed by two main approaches for the evaluation of mHealth apps available for PwD healthcare and their carers.

- The first approach is the comprehensive review of existing literature about mHealth apps used for dementia healthcare.
- The second approach is an identification of existing mHealth dementia apps that are commercially available in smartphone stores.

### 3.3. Literature Search and Selection Approach

#### 3.3.1. Literature Search Strategy

To identify relevant previous literature regarding mHealth apps in dementia healthcare, a keyword search was performed on five electronic databases:* MEDLINE*®*, PubMed, SAGE, IEEE Xplore*, and* Science Direct*. Search terms used in all databases were* “dementia AND mobile apps,” “dementia AND mobile technology,” “dementia AND smartphone,” “dementia AND tablet,” “android app AND dementia,” “iOS app AND dementia,” “mHealth app AND dementia,” “mobile health app AND dementia,” “dementia AND caregivers AND mobile app,” *and* “dementia AND carers AND mobile app.”* The hits of keywords in scientific databases are listed in Table 2.

#### 3.3.2. Literature Inclusion and Exclusion Criteria

**Figure 2:**
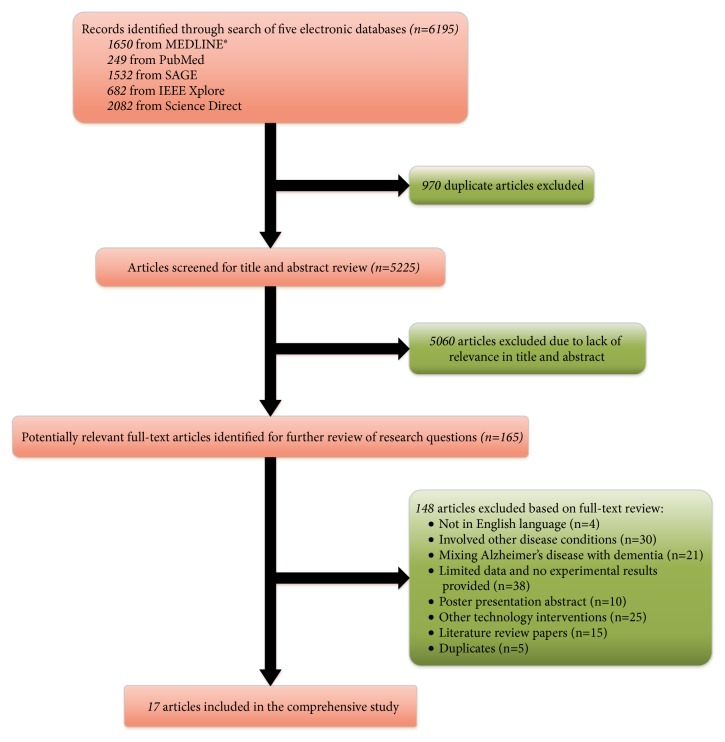
Flow diagram of literature selection.

In this comprehensive study, we included peer-reviewed, full-text articles published in English during last five years (from 2014 to 2018) and aimed to identify such studies that specifically assessed the implementation of using mHealth based apps on PwD and the caregivers. The initial searches based on keywords and search terms yielded a total of 6195 research articles including 1650 from MEDLINE®, 249 from PubMed, 1532 from SAGE, 682 from IEEE Xplore, and 2082 from Science Direct. The first step after the initial search was an exclusion of duplications, and 970 articles were excluded. Remaining research articles were screened by their title and abstract. We excluded 5060 articles due to lack of relevance in the title and/or abstract. After applying inclusion/exclusion criteria at the abstract level, 165 full-text articles were reviewed to check the appropriateness for inclusion; see Figure 2 for inclusion criteria. For the full-text articles, we rejected 148 that did not match with our interest scope. We shortlisted 17 publications reports on the intervention of dementia apps. These included studies piloted and evaluated for their effects on dementia patients and their carers. All these shortlisted articles recorded useful information regarding mHealth applications in dementia healthcare; therefore, these are included in the review.

#### 3.3.3. Literature Data Synthesis

After the shortlisting of research articles, the data is extracted and systematically organized on the spreadsheet. It is then compared to find out correlations between studies. This process results in an identification of categories and subcategories of mHealth applications for PwD and their caregivers as shown in Table 3.

### 3.4. mHealth Based Dementia Applications Search and Selection Approach

#### 3.4.1. App Search Strategy

During July 2018, the keyword search of mHealth based dementia applications was performed on two most common commercial stores for smartphones: Google Play Store (developed by Google LLC, for Android OS devices) and App Store (developed by Apple Inc., for iOS devices). The devices used for searching the terms are LG Nexus 5 (Android OS device) and iPhone 4s (iOS device). Initially, the search terms for both aforementioned stores were same as the keyword search terms of literature section (see Section 3.3.1. for details), but these terms generated maximum unrelated results, so the simple search term dementia was used to get more disease-specific app searches.

#### 3.4.2. App Inclusion and Exclusion Criteria

The initial search based on dementia keyword yielded a total of 356 results, including 106 in iOS and 250 in Android. After removing the duplicates, we screened names, ratings, and prices of apps generated from search keyword, as these are the prominent available features at the search result screen of two selected app stores. At first stage, a total of 58 applications were excluded from both mobile app stores due to lack of compliance with our inclusion criteria 1 and 6; see Table 4 for details of inclusion and exclusion criteria. AsiOS App store does not show download counts, so we included apps as per the inclusion criteria 1-4 of Table 4. The complete inventory of shortlisted commercial apps is listed in Table 5. In comparison to generic mHealth apps, the availability of mobile apps geared towards dementia healthcare for both PwD and their caregivers is relatively sparse with 35 apps by applying inclusion/exclusion criteria.

**Figure 3:**
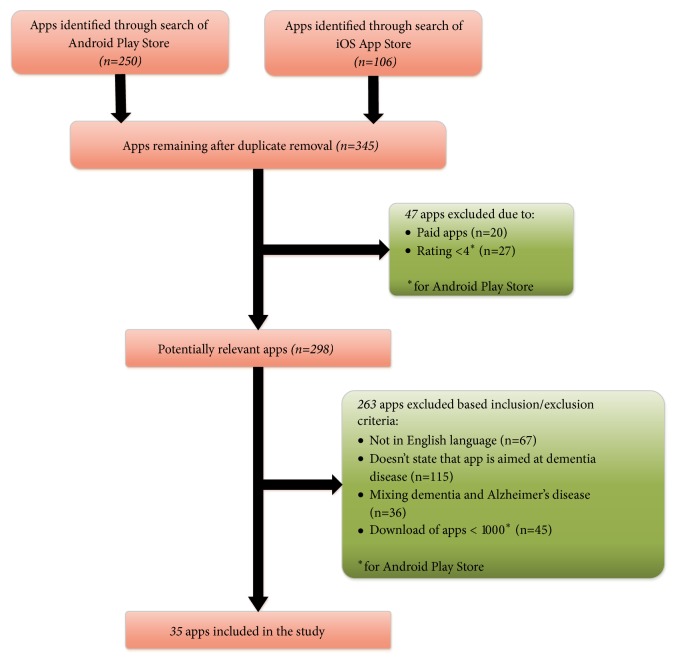
Selection process of mHealth based dementia apps.

The selection process of mHealth based apps for dementia care is illustrated in Figure 3. After the shortlisting of apps, it is observed that existing apps generally addressed different dementia specific aspects which are overlying with the identified categories of mHealth apps in research articles (see Section 3.3.3 for categories and subcategories details).

## 4. Results

### 4.1. Literature Review of mHealth Apps Used in Dementia Healthcare

Of the seventeen (17) included articles in this review, seven (07) articles involved tablet assistance, two (02) focused on using only mobile devices, and seven (07) research articles used both tablets and mobile devices for mHealth. However, one study used a smart watch device with the smartphone.

All cited studies used smartphones and tablets running iOS and Android operating systems. Regarding the categories of studies as mentioned in Table 3, five (05) studies piloted and evaluated the usability of mHealth applications from cognitive training and daily living category [40–44], five (05) studies were from screening of dementia category [45–49], one (01) study combined cognitive training and screening of dementia categories [50], three (03) studies were from health and safety monitoring category [51–53], two (02) studies were from leisure and socialization category [54, 55], and one (01) study covered navigation section [56]. The data extracted from research articles are study, study type, study aim, study methodology, technology type (i.e., mobile, tablet, etc.), participants in the study, location, study outcome, application name, application features, and category of study as shown in Table 6.

*Category 1: Cognitive Training and Daily Living*. The first category outlines different strategies to help cognitively impaired dementia people. The included studies in this category identified six (6) subcategories, i.e., memory, communication, logical thinking, attention, language abilities, and schedule. Three studies [40–42] used smart devices (i.e., tablets, smartphones, and smart watches) with already installed applications. Kong et al. [40] conducted sessions for PwD to address four major cognitive domains, i.e., language, visual-spatial, memory, and problem-solving. The participants rated apps on a scale of 1 to 5 at the end of the session. This exploratory study stated that apps with simple activities are more usable, whereas an app with confusing directions and complex vocabulary resulted as least usable among PwD. Likewise, other user-centred design methodologies [41] addressed communication and scheduling cognitive domains by including smart watch and smartphone device to support dementia people and their caregivers. This study concluded that cognitive training based mHealth apps provide a promising environment for PwD to perform better in their daily routine, whereas orientation feature has more usability and usefulness to carers. Vahia et al. [42] also investigated the feasibility, usability, and safety of tablet apps in managing agitated behavioural symptoms among PwD. The 70 installed apps are reviewed and rated as per complexity score ranging from 0 (less complex) to 10 (highly complex). The results of this open-label study demonstrated the positive impact of the tablet as a nonpharmacological intervention for reducing agitation among old dementia patients.

Other three included studies [43, 44, 50] developed applications for engaging PwD in different cognitive exercises. Dethlefs et al. [43] used cognitive stimulation technique to involve dementia people in cognitive activities and social functioning, i.e., the conversation about childhood, puzzles, or memory activities. This study used “Wizard-of-Oz” setup to support elderly people with CDR less than 3. The experiment involved human wizards (informal carers) with previous experiences of providing cognitive stimulation on a human-to-human basis. The human wizard was responsible to understand the natural spoken language of the dementia patient and respond through prespecified text templates provided by an app. The off-the-shelf text-to-speech software converted the text response of wizards into speech. Boyd et al. [50] developed EnCare diagnostics (ECD) app and brain fit plan (BFP) app and evaluated the usability on healthy and cognitively and physically impaired older adults. The EnCare diagnostics (ECD) app is used to access the cognitive state of individual (discussed in screening category) while brain fit plan (BFP) is used to suggest PwD different tasks to perform daily. Tyack et al. [44] performed an exploratory study to check three cognitive domains, i.e., stimulation, remembering, and attention of PwD. They developed an art viewing app to display paintings and photographic images of historic places. The quantitative and qualitative findings of study approved touchscreen-based art interventions as well-being benefits for PwD.

*Category 2: Screening*. Screening technology is intended to help elderly people by giving them an early awareness of any symptoms that may lead to cognitive impairment. It is also helpful for carers by identifying the risks of dementia among their family members. Dementia detection and cognitive screening based subcategories emerged from the selected articles. Two articles [46, 49] are focused on predicting the risk of dementia among people by using the mobile app. The earliest of these two articles [46] developed an app to predict the late-life dementia risks based on CAIDE (Cardiovascular Risk Factors, Aging, and Incidence of Dementia) risk score, whereas Shibata et al. [49] developed an mHealth app with natural language processing (NLP) technique to detect the dementia symptoms as the change in language ability is considered as one of the early signs of having dementia. The app examines the free narrative speech of a person and calculates four language abilities such as the number of words in speech segment (tokens), the number of different words in speech segment (types), type-token ratio (TTR), and potential vocabulary size (PVS). The results validated significant difference in language ability between PwD and healthy elderly people.

Four (04) studies [45, 47, 48, 50] designed and developed cognitive screening of dementia apps. MOBI-COG app [45] allowed people to take dementia screening tests at home by incorporating convenient, automated, and easy measures to administer features. The user is asked to perform three tasks, i.e., remembering words, clock drawing, and recalling words and at the end displays an automated assessment of these tasks. The experimental setup involved normal people while the other three included articles [47, 48, 50] included dementia people to validate the effectiveness of mobile app as a screening application. Zorluoglu et al. [47] proposed a mobile cognitive screening (MCS) app. This app featured 33 tests in fourteen (14) different types for the assessment of cognitive functions. The eight (08) targeted cognitive functions are memory, attention, language, orientation, arithmetic, visual, abstraction, and executive functions. To check the effectiveness of MCS test, all participants in this study are tested by using this app and Montreal cognitive assessment (MoCA)—an eminent assessment technique for cognitive screening—to compare the results of two tests. The scores of the same participants revealed the correlation between MCS and MoCA score. Another study [48] also used touchscreen tablet-based mHealth app to identify early cognitive impairment in older adults. This study concluded that the cognitive battery test is a cost-effective solution for the screening of early MCI and AD among dementia people. However, Boyd et al. [50] developed EnCare diagnostics app to access the cognitive state of individuals with dementia. The app is proposed as an alternative to the mini-mental state examination (MMSE) which is another well-known cognitive screening test. The participants of this study stated limitations in using BFP, a cognitive training based mHealth app as explained earlier in category 1, but they showed a positive response in using ECD.

*Category 3: Health and Safety Monitoring*. The third category incorporates the monitoring of health and safety conditions of dementia patients. Based on our included studies, this category is subdivided into two subcategories: fall detection and emergency help. For emergency situations, two apps [52, 53] help in assessing the pain of elderly dementia patients. Pain Assessment app [52] is designed and developed on the basis of the Abbey pain scale (a one-minute pain assessment questionnaire for people with severe dementia), whereas PainChek™ [53] is also used to access the pain by incorporating 42 items from 6 different domains (face, voice, movement, behaviour, activity, and body). Smartphone camera at front-end and artificial intelligence (AI) algorithm at the back-end are used to detect the facial expressions of dementia patient from already classified nine (09) facial expressions. The other five (05) domains required manual input from the user. After the completion of input in six different domains, the app generates a numerical pain score ranging from no pain (Score: 0-6) to severe pain (Score ≥16). These studies postulated that mHealth tools are useful in the prehospital settings and emergency situations and these apps have a potential to transform the pain assessment of PwD who are unable to communicate and express their feelings to others.

On the other hand, Bayen et al. [51] provided safety monitoring app to detect the falls of a dementia patient in a care facility. The experimental setup used wall-mounted cameras to record the falls and smartphone device for continuous checking of PwD. The smartphone-based app featured live video view from mounted cameras and video review service to staff of care facility to ensure the better quality of care provision. This observational pilot study concluded that video review facility is helpful in screening the severity of falls and injuries occur due to falls. The study also highlighted that video replay can be helpful in identifying deficiencies of cognitive behaviour and environmental circumstances contributing to the fall.

*Category 4: Leisure and Socialization*. This category “leisure and socialization” of included studies provides reminiscence and socialization therapy to PwD. mHealth based app InspireD [54] investigated the effects of reminiscence activity on PwD. The main functions of the app are the following: (1) to bring people living with dementia and their caregivers together and (2) to store generic and personalized memorabilia in the form of audio, video, and photos. This feasibility study showed that PwD interacted with this app more than their caregivers in reminiscence section. Additionally, dementia people preferred to view personalized multimedia content than generic videos or photos. Welsh et al. [55] designed and developed mobile and tablet-based app Ticket to Talk to promote intergenerational interactions between young relatives and dementia individuals. The application is evaluated through the trials of two families, a care home and an elder people group. The thematic analysis of obtained data from participants revealed that Ticket to Talk promotes and manages reminiscence and helped young people to start and maintain a conversation.

*Category 5: Navigation*. The last category relates to providing “navigation facility” and it is very useful for caregivers as they can track the location of patients. A pilot study [56] presented the mobile-based locating system with four major functions: locating, call, alarm, and hotline, and two subfunctions: zone mapping and zone sharing. The outcomes of the proposed application are measured in terms of usability and rating of app features. The usability score decreased over time due to technical deficiencies of a prototype system. Regardless of the technical discrepancies, it is noticed that PwD and their caregivers are more receptive about the assistance of mHealth technology in home dementia care. It is also found out that male caregivers were more willing to purchase this prototype than female caregivers.

### 4.2. Existing mHealth Based Commercial Apps Used in Dementia Healthcare

Out of 35 identified mHealth dementia apps, eleven (11) apps belong to cognitive training and daily living category, four (04) apps are from screening category, four (04) apps are from health and safety monitoring category, six (06) apps are from leisure and socialization category, one (1) app is from navigation category, and nine (09) apps are for the support of carers. The following data are extracted from apps: app name, app release year, app rating (if any), app review count (if any), platform, the app offered by, app category, privacy policy (provided or not), and features of apps. The details of identified apps from Android and iPhone platforms are mentioned in Table 7.

## 5. Discussion

mHealth has become the fast-growing assistive technology that supports the broad range of healthcare services including mental healthcare [57]. The mHealth applications for dementia are divided into two broad categories:

1. Apps used by dementia sufferers for the improvement in their quality of life
2. Apps used by the health care providers (health professionals or informal caregivers) to perform a variety of functions or services for PwD

The aim of this comprehensive study is to identify, appraise, and synthesize the existing evidence of using mHealth based dementia apps as a healthcare resource for people with dementia. Secondly, this study also contributes to finding out the influence of mHealth apps in supporting informal caregivers. In this study, seventeen (17) articles are reviewed and a total of thirty-five (35) apps are shortlisted. All included articles in this comprehensive study provided a clear description of the experimental methodology and outcomes of using mHealth apps for dementia people and their caregivers. The systematic data analysis of included studies and review of commercial apps resulted in the identification of five categories and subcategories of mHealth based dementia applications. These categories of dementia mobile applications can help in finding out the existing apps and planning of new mHealth based interventions for PwD and their carers. Regarding the participants of the included studies, seven (07) studies recruited PwD and their professional and informal carers, five (05) studies engaged PwD only, three (03) studies involved control groups (healthy older people) and PwD, one (01) study performed experimental study on healthy adults, and one study conducted a usability study on care professionals.

All articles are evaluated as providing useful and relevant information regarding mHealth apps in dementia care yet there were some limitations in the studies. Majority of these studies were focused on testing the feasibility and usability of existing or developed mobile applications for PwD. However, only one study adopted a user-centered design methodology in development of mobile application by involving dementia people. User feedback in a systemic manner can deliver an effective application as few studies highlighted the reluctance of moderate to severe CDR people in using assistive technology due to complex features of applications. Other than this, the most apparent gap in the literature is the lack of awareness or training applications for informal caregivers but the exploration of commercially available apps acknowledged in the availability of applications to support caregiver by providing knowledge about dementia healthcare.

This review concluded that the convenience and uniformity of using mHealth based dementia apps make it likeable to use by dementia people in daily routine. It is also validated that smartphone-based assistive technology is psychometrically valid in evaluating the cognitive skills of dementia people. The adoption of mHealth apps among dementia people is highly correlated with the simple features of apps and can be used as a nonpharmacological intervention. The gradual improvement in the well-being of dementia people in daily living is witnessed by using mobile apps. The impact of well-being manifests in the form of their behaviour, emotion, cognition, and relationship with carers.

It is found out that informal care places a high amount of mental, emotional, physical, and financial stress to the carers. Using a mobile app with navigation feature makes it possible to address one of the significant stress factors for carers, i.e., the disorientation of PwD while outside the environment. Similarly, the video monitoring-based applications have the tendency to provide a promising assistive tool to support carers. These apps with video monitoring help carers to detect any falls or accidents of PwD in daily living to prevent them from serious injuries. The care is less burdensome when carers have information and education about dementia so that the health-related problems of PwD are well managed and well addressed. Mobile apps make pain assessment process of dementia people simpler and easier. The tools are useful in emergency situations and have the capability to transform the pain of PwD especially for those who are unable to communicate or express their feelings to others. Many mHealth based apps are now working on understanding the language abilities of PwD so that the provision of better care and quality of life can be ensured. Lastly, this review also recommends that the quality of life of PwD can also be improved by using reminiscence therapy and socialization among family members and dementia people. It can be achieved by using such mHealth based dementia apps that engage dementia people in a series of daily living activities and provide carers with a starting point to make conversation with the dementia sufferer.

### 5.1. Limitations

In this paper, only studies published in English during the last five years (from 2014 to 2018) of five databases were included which may not take account of papers published in other languages and other years.

## 6. Conclusion

The purpose of this comprehensive study is to identify, evaluate, and synthesize the existing evidence on the use of mHealth based applications (apps) as a healthcare resource for people with dementia and assistance to carers. A review of both peer-reviewed and full-text literature was undertaken along with mHealth based dementia applications from commercial mobile stores. The results of this review have identified five major areas of mHealth based dementia applications. To date, applications for dementia healthcare are used in cognitive training, screening, health and safety monitoring, leisure and socialization, and navigation. The cognitive training and leisure and socialization of PwD are attained by engaging them in a variety of activities in daily living such as memory, attention, logical thinking, scheduling, and communication. For informal caregivers, the applications are focused on providing awareness and education about dementia. The carer support in the form of navigation and health and safety monitoring is also noticeable in mobile applications. The purpose of mobile applications is to assist carers in understanding the dementia people with the objective of providing a better quality of life. This review concluded that mHealth based assistive technology has the potential to provide efficient healthcare facility to PwD and support caregivers as this technology provides simple interactive features and promotes independence.

## Figures and Tables

**Table 1:** Categories of dementia and level of memory impairment [21, 23].

Dementia Type	Memory Impairment Level
0 (Normal)	No mental abnormality but has a minor degree of memory impairment.
1 (Mild)	Definite memory impairment and calculating ability.
2 (Moderate)	Definite memory impairment, but also disorientation for time and/or place.
3 (Severe)	Definite memory impairment, but also the difficulty of self-care.

**Table 2:** Keywords hits in scientific databases.

Search Keywords	MEDLINE®	PubMed	SAGE	IEEE Xplore	Science Direct
dementia AND mobile apps	259	50	75	60	466
Dementia AND mobile technology	388	46	592	45	350
dementia AND smartphone	127	31	112	48	80
dementia AND tablet	114	89	380	15	172
android app AND dementia	47	5	47	10	75
iOS app and dementia	158	5	43	7	15
mHealth app AND dementia	151	5	16	437	44
mobile health app AND dementia	137	5	10	35	48
dementia AND caregivers AND mobile app	127	4	102	10	432
Dementia AND carers AND mobile app	142	9	155	15	400

**Table 3:** Categories and subcategories of mHealth based dementia applications.

Categories	Subcategories
Cognitive Training and Daily Living	Memory
	Communication
	Logical Thinking
	Attention
	Language Abilities
	Schedule

Screening	Dementia Detection
	Cognitive Screening

Health and Safety Monitoring	Fall Detection
	Emergency Help

Leisure and Socialization	Reminiscence Therapy
	Socialization Therapy

Navigation	Tracking
	Location Service

**Table 4:** Inclusion and exclusion criteria for mHealth based dementia applications in Android Play Store and iOS App store.

Inclusion Criteria	Exclusion Criteria
(1) App availability in English language.	(1) App un-availability in English language.
(2) Free app.	(2) Paid apps.
(3) States aimed at dementia disease.	(3) Doesn't state that app is aimed at dementia disease.
(4) Developed in support with research centre/piloted at dementia care homes/Popular or award-winning.	(4) Mixing dementia and Alzheimer's disease.
(5) Downloads of app ≥ 1000^*∗*^ if app not fulfilling criteria 4.	(5) Downloads of app < 1000^*∗*^.
(6) Rating ≥ 4^*∗*^ if app not fulfilling criteria 4.	(6) Rating < 4^*∗*^.
	(7) Duplicate app.

^*∗*^For  *Android apps only*.

**Table 5:** Inventory of mHealth based dementia applications in Android Play Store and iOS App store.

App Name	Platform	App Link
(release year)		
SingFit (2011)	iOS	https://itunes.apple.com/us/app/singfit/id442827581

Constant Therapy (2012)	iOS	https://itunes.apple.com/us/app/constant-therapy/id575764424

Pain Rating Scales (2015)	Android	https://play.google.com/store/apps/details?id=com.etz.painassessment

Brain Training Journey (2015)	iOS	https://itunes.apple.com/us/app/brain-training-journey/id968590641

Care Tracker (2015)	Android & iOS	https://play.google.com/store/apps/details?id=com.stellacare.stella
		https://itunes.apple.com/us/app/care-tracker/id914880113

Dementia Care Matters (2015)	iOS	https://itunes.apple.com/us/app/dementia-care-matters/id1002340094

Dementia Test-Risk Calculator of dementia (2015)	iOS	https://itunes.apple.com/us/app/dementia-test-risk-calculator-of-dementia/id1014958634

Memory Box (2015)	Android & iOS	https://play.google.com/store/apps/details?id=com.sci.memorybox
		https://itunes.apple.com/us/app/memory-box/id989093504

Dementia/ Digital Diary/Clock (2015)	Android	https://play.google.com/store/apps/details?id=com.fashmel.alzclock

Dementia Clock (2015)	Android	https://play.google.com/store/apps/details?id=com.wearingthegreen.pc.otdementiaclock

Care4Dementia (2015)	iOS	https://itunes.apple.com/pk/app/care4dementia/id1029281368

GreyMatters: Beyond Dementia (2015)	iOS	https://itunes.apple.com/us/app/greymatters-beyond-dementia/id900645661

Living and Dying Well With Dementia (2015)	iOS	https://itunes.apple.com/us/app/living-and-dying-well-with-dementia/id945365520

Consult Geri: Dementia (2015)	iOS	https://itunes.apple.com/us/app/consultgeri-dementia/id962437779

Care and Connect: Dementia Friendly Places (2015)	iOS	https://itunes.apple.com/gb/app/care-and-connect-dementia-friendly-places/id985980528

Sea Hero Quest (2016)	Android & iOS	https://play.google.com/store/apps/details?id=com.glitchers.seaheroquestvrdaydream
		https://www.oculus.com/experiences/gear-vr/1639295696089857/

Jigsaw Puzzle: Spider (2016)	Android	https://play.google.com/store/apps/details?id=com.cocopapasoft.spiderpuzzle

Dementia Risk Tool (2016)	Android & iOS	https://play.google.com/store/apps/details?id=com.dementiarisktool
		https://itunes.apple.com/us/app/dementia-risk-tool/id1160712531

Dementia guide for carers and care providers (2016)	iOS	https://itunes.apple.com/gb/app/dementia-guide-for-carers-and-care-providers/id1085813885

MindMate-Health aging (2016)	iOS	https://itunes.apple.com/us/app/mindmate-for-a-healthy-brain/id1030422375

BrainScore: Connect Dots (2016)	iOS	https://itunes.apple.com/us/app/brain-score-connect-dots/id1146159421

A walk through Dementia (2016)	Android & iOS	https://play.google.com/store/apps/details?id=com.alzheimersresearchuk.walkthroughdementia
		https://itunes.apple.com/us/app/a-walk-through-dementia/id1242267344

Folstein Test (2016)	Android	https://play.google.com/store/apps/details?id=appinventor.ai_openmylabfeed.MMSEDemo

My House of Memories (2016)	Android	https://play.google.com/store/apps/details?id=com.nml.myhouseofmemories

Dementia Caregiver Application v2 (2016)	iOS	https://itunes.apple.com/us/app/dementia-caregiver-application-v2/id1068601934

myPlayLife (2017)	iOS	https://itunes.apple.com/us/app/myplaylife/id1205956380

Brain Test (2017)	iOS	https://itunes.apple.com/us/app/braintest/id1030864182

CogniCare-Enabling personalized dementia care (2017)	Android	https://play.google.com/store/apps/details?id=com.cognihealth.cognicare

REMEMBER ME (2017)	Android	https://play.google.com/store/apps/details?id=com.cuzzrek.rememberme

Alzheimer's Speed of Processing Game-ASPEN (2017)	Android	https://play.google.com/store/apps/details?id=com.TinyHappySteps.Bird

Cognitive Rehab in Dementia (2017)	Android & iOS	https://play.google.com/store/apps/details?id=com.connectinternetsolutions.nes.dementia
		https://itunes.apple.com/gb/app/cognitive-rehab-in-dementia/id1316426480

eSLUMS (2017)	iOS	https://itunes.apple.com/us/app/eslums/id1141690870

SymptomGuide™ Dementia (2018)	iOS	https://itunes.apple.com/us/app/symptomguide-dementia/id1357298369

Dementia Respond (2018)	Android	https://play.google.com/store/apps/details?id=com.appmakr.dementiarespond

Your Care Card (2018)	iOS	https://itunes.apple.com/us/app/your-care-card/id1355112732

**Table 6:** Summary of reviewed studies.

Study (year and country)	Study Type	Study Aim and Methodology	Participants^1^	mHealth app with Features	Study Outcomes	Study Category
Nirjjon et al. [45], 2014, USA.	Usability Study	Aim: To provide convenient, automated and easy to administer dementia screening test that can be taken at home. *Methodology* (i) Development of application. (ii) Use local database for displaying words on the screen. (iii) Pre-processing, feature extraction, training and classification for clock drawing test. (iv) k-NN algorithm for classification of recognized digits.	7 healthy adults (3 females, 4 males) age ~ 25-35	*MOBI-COG* App with remembering words, clock drawing test and recalling words.	Questionnaire of the usability study, 99.53% accuracy in clock drawing test.	*Screening*

Kong [40], 2015, USA.	Exploratory Study	Aim: To explore the implementation of apps on the iPad to conduct cognitive exercises. *Methodology* (i) Keyword based app selection from iTunes. (ii) Questionnaire to determine the familiarity of the participant with technology. (iii) Finalize the apps based on a questionnaire.	10 PwD (6 males, 4 females) age ~ 64-84 MoCA: 15.74 (diagnosed with early-stage dementia)	*Free Apps (iTunes)* with visual-spatial, memory and problem-solving.	Participants' rating of exercise, Clinicians response for further implementation of apps.	*Cognitive training and daily living*

Zorluoglu et al. [47], 2015, Turkey.	Novel Study	Aim: To evaluate the cognitive skills by involving different tests for cognitive functions assessment. *Methodology* (i) Experimental study to conduct 33 tests in 14 different cognitive skills. (ii) Display the radar chart to show progress.	23 participants with CG+PwD *Control Group* 9 healthy people age ~ 63-95 Females = 78.78%, Males= 22.22% Avg. MoCA score: 24.55 ± 3.08 *PwD Group* 14 dementia people age ~ 73-89 Females = 78.57%, Males= 22.43% Avg. MoCA score: 13.57 ± 5.61	*Mobile Cognitive Screening (MCS) App* with trail making, clock drawing, attention, visual, shape similarity, shape matching, arithmetic, proverb, naming, numbers, colourful shapes, market, date, and story recall test.	Correlation between MCS and MoCA scores was r^2^=0.57; this correlation was meaningful (p <0.01).	*Screening*

Sindi et al. [46], 2015, Finland.	CAIDE Study	Aim: To predict the risk of dementia through CAIDE (cardiovascular risk factor, ageing and incidence of dementia) Risk Score app. *Methodology* (i) Development of app based on CAIDE dementia risk score by involving information on age, educational level, hypertension, hypercholesterolemia, obesity, and physical inactivity.	Four independent population-based random samples Participants MMSE score ≤ 24	CAIDE Risk Score App with graphical display of risk scores, risk of developing dementia estimation, guidance and suggestions to users, the recommendation of a health practitioner.	Receiver operating characteristics (ROC) curve demonstrated the dementia risk score predicted effectively. 95% confidence interval (0.71-0.83).	*Screening*

Thorpe et al. [41], 2016, Denmark.	UCD case	Aim: A UCD (User-centred design) to investigate the adoption of pervasive assistive technology among mild dementia people. *Methodology* (i) Selection of key support features. (ii) User testing of selected applications.	5 Dyads with PwD of MCI PwD: 3 males, 2 females age~ 61-73	*11 Free Android Apps* with scheduling, navigation, communication, emergency help, monitoring and orientation.	System Usability Scores (SUS) in usability testing and field testing.	*Cognitive training and daily living + Navigation + Health and Safety Monitoring*

Vahia et al. [42], 2016, USA.	Open-Label Study	Aim: To investigate the feasibility, usability and safety of tablet apps in managing agitation among dementia patients. *Methodology* (i) Personalization of tablet applications (rate selected apps as per complexity). (ii) Find the correlational analysis between tablet use and demographic characteristics of the group.	36 participants (n) with Mild to Severe dementia (females: 61.1%, males: 38.9%) Mildly impaired MoCA score: 18 to 25; n=23 Moderately Impaired MoCA score: 10 to 17; n=7 Severely Impaired MoCA score <10; n=16 Age~ avg 79.9	*70 Free iTunes Apps* with communication, games, music and musical instruments, video, entertainment and recording, web browser and news, and picture/photograph-viewing apps.	Well Utilization of app. Agitation reduction in the mildly impaired group as compared to severe impaired = p =0.02.	*Cognitive training and daily living*

Boyd et al. [50], 2017, Ireland.	Usability Study	Aim: To develop and evaluate the usability of EnCare diagnostics (ECD) and Brain Fit Plan (BFP) app. *Methodology* (i) Select Android apps. (ii) Design trails-based study. (iii) Usability assessment of apps.	Includes 02 trials: *Trial 1*: 11 healthy people (age > 60) Females: 9, Males: 2 4 older adults with physical impairment *Trail 2*: 8 older adults Females: 4, Males: 4 MMSE score: 21-26 Diagnosed with early-stage dementia	*ECD App* with screening of dementia. *BFP App* with logical thinking based on existing apps (chess, jigsaw etc.).	75% dropout at Trail 1 for BFP testing due to in familiarity with technology and lack of carer support. High acceptability in ECD. No usability issue in ECD.	*Screening (ECD)* *Cognitive training and daily living* *(BFP)*

Dethlefs et al. [43], 2017, England.	Pilot Study	Aim: To provide elderly people and PwD computer-based cognitive simulation via spoken natural language. *Methodology* (i) Wizard-of-Oz interface. (ii) To process input speech of participant and respond accordingly.	23 participants with Healthy and PwD *Healthy People: 13* Avg. age~ 84.33 *PwD: 10* Avg. age~ 78.20	*Wizard-of-Oz System* with sorting, naming, recall, quiz and proverb.	Enjoyment and wanted to use it again.	*Cognitive training and daily living* *(Memory & Communication)*

Bayen et al. [51], 2017, USA.	Pilot Study	Aim: To analyse how continuous video monitoring and review of falls of PwD can support a better quality of care. *Methodology* (i) Measure the count of falls occurring in the video covered area. (ii) Evaluate the use of video recording and replay possibilities for care practice.	Overall 38 PwD with 10 participants allowing written and oral consent for video recording in their private rooms.	*SafetyYou App* with video view feature from past 72 hours, live video view on a smartphone from each installed camera.	Drop in fall rate. Positive impact on quality of care.	*Health and Safety Monitoring* *(Fall Detection)*

Mulvenna et al. [54], 2017, England.	Feasibility Study	Aim: To investigate the effects of individual-specific reminiscence activity provided by using the app. *Methodology* (i) An agile development approach for prototype (ii) Local database for the storage of multimedia content.	28 Dyads	*InspireD App* to enable PwD and their carer gather together, store selected generic and personalized memorabilia.	Engagement. Use for personal multimedia content.	*Leisure and Socialization (Reminiscence Therapy)*

Megges et al. [56], 2017, Germany.	Pilot Study	Aim: To evaluate the user experience regarding prototype locating system in home dementia care to better understand the needs and preferences of PwD and their carers. *Methodology* (i) Use of locating system for 4 weeks. (ii) Measure usability, product functions and features. (iii) Measure caregiver burden perceived self-efficacy, the frequency of use, and willingness to purchase the prototype.	18 Dyads *PwD Count* 3 MCI, 6 Mild disease severity, 9 moderate disease severity Avg. age ~ 71.94 *Carers Count* 10 husbands, 6 wives, 2 daughters	Prototype Locating System with a call, alarm, location tracking and service hotline. *Sub-Features*: Zone mapping Zone Sharing	Usability rating declined, product functions rated positively. No difference in caregiver burden and perceived self-efficacy. Moderate frequency of use, and high willingness to purchase.	*Navigation*

Tyack et al. [44], 2017, England.	Quantitative and Qualitative Study	Aim: To explore whether art-based intervention can be delivered via a touchscreen tablet device displaying art images. *Methodology* (i) The quantitative data followed a quasi-experimental repeated measures design. (ii) Compare before and after use of the tablet on well-being. (iii) The qualitative data collected during interviews was analysed using thematic analysis.	12 Dyads *PwD* Males: 8, Females: 4 Avg. age ~ 75 Diagnosed dementia with 4 years *Carer* Males: 2, Females: 10 Avg. age ~ 66	Art Viewing App for presenting choices of art genre to view from museums and area artists.	Improvement in well-being and enthusiasm.	*Cognitive training and daily living* *(Stimulating, Remembering, Attention)*

Docking et al. [52], 2017, England.	Qualitative Study	Aim: To improve the pain assessment of PwD and management in this vulnerable population. *Methodology* (i) conducting usability testing of an app to assess pain in adults with dementia treated by paramedics. (ii) evaluate the appropriateness of the algorithm used in an app. (iii) identifying areas for further development and response to recommendations.	24 paramedic students from two England Ambulance services. Females: 11, Males:13 7 Delphi Panel Experts Females:1, Males: 6	*Pain Assessment App* with the Abbey Pain Scale based questionnaire.	Positive usability testing. Paramedics Students and Delphi panel of experts state it as a useful tool in a pre-hospital setting.	*Health and Safety Monitoring* *(Emergency Help, Pain assessment)*

Huang et al. [48], 2017, Taiwan.	Experimental Study	Aim: To develop a psychometrically valid touchscreen tablet-based cognitive test battery to identify early cognitive impairment due to dementia in older adults. *Methodology* (i) To conduct 8 tests with 13 subscores for the measurement of different cognition skills.	120 participants (n) CDR=0; n=41; age ~64-88 CDR=0.5 with MCI; n=43; age ~59-91 CDR=1; n=36; age ~56-91	Attention, Visual Memory, Visuospatial, Reaction Time, Fine Motor Control. (App name not mentioned)	Mann–Whitney U tests were significant for CDR = 0 versus 0.5, and CDR = 0 versus 1. Confirmation of the four-factor model by Confirmatory factor analysis (CFA).	*Screening*

Welsh et al. [55], 2018, England.	Experimental Study	Aim: To encourage conversation between younger people and their old relatives living with dementia. *Methodology* (i) Extend the previous work by developing an application that incorporates the following four design requirements: (1) Setting up a profile. (2) Inspiration (media to use for talk). (3) Collecting media. (4) Preparing a conversation.	Deployment with 02 families of PwD. Deployment in care home involves 3 volunteers to maintain a conversation with 10 residents, age ~ 80-95 with moderate to severe dementia. 9 older people with personal/professional dementia experience for expert critique.	*Ticket To Talk App* to create a profile for an older person, prompt carers to collect media (audios, videos, and images etc.), helps carers to organize media, use media as prompts and conversation starters	Promoting and managing reminiscence, Starting and maintaining conversation, Redistributing Agency.	*Leisure and Socialization (Reminiscence Therapy, Relaxation)*

Shibata et al. [49], 2018, Japan.	Experimental Study	Aim: To report that MCI patients have a significantly larger vocabulary than healthy elderly people. *Methodology* (i) Use automatic speech recognition for measuring language abilities. (ii) Analyze narrative speech and find four language abilities scores.	18 participants PwD: 8 with MCI CG: 9	*VocabChecker App* to calculate the number of tokens, number of types, token type ratio, potential vocabulary size.	p-values in t-test show that token type ratio of PwD is higher than CG (p <0.05, highly significant)	*Screening (language abilities based)*

Atee et al. [53], 2018, Italy.	Novel Study	Aim: To describe a novel system called PainChek™ focusing on its conceptual foundation, clinical and technical contents, clinical use, and practical tips for use in clinical settings. *Methodology* (i) Following things to consider while developing an application: (a) Subjective nature of pain. (b) Complexity, and dynamicity of pain as a construct. (c) An objective description of key pain behaviours. (d) The temporality of pain and related behaviours. (ii) Simple scoring mechanism.	12 Dyads PwD Males: 8, Females: 4 Avg. age ~ 75 Diagnosed dementia with 4 years Carer Males: 2, Females: 10 Avg. age ~ 66	*PainChek*™* App* to test pain scale (42 items across 6 domains), to display pain assessment log, Pain chart and local patient database, to provide medications and therapies and comments section.	Sound psychometric properties. Excellent concurrent validity, interrater reliability, internal consistency, and excellent test re-test reliability.	*Health and Safety Monitoring* *(Emergency Help, Pain assessment)*

^1^ PwD: People with Dementia; CG: Control Group; MoCA: Montreal Cognitive Assessment; Dyad: PwD and carers; MMSE: Mini-Mental State Examination; MCI: Mild Cognitive Impairment.

**Table 7:** List of mHealth based dementia applications in Android and iPhone platforms.

App Name (release year)	Offered by	Platform	Features	Category	Privacy Policy Mentioned (Yes/No)	Downloads/Reviews (Raring), If any
SingFit (2011)	Medical Health Technologies	iOS	(i) Provides music therapy.	Leisure and Socialization	No	63 reviews (4.0)

Constant Therapy (2012)	Constant Therapy, Inc.	iOS	(i) Use personalized exercises at home (ii) Get up to 4 times more practice with immediate feedback. (iii) Pairs perfectly with clinic cognitive speech therapy.	Cognitive Training (Communication, Language Abilities)	Yes	300 reviews (4.5)

Pain Rating Scales (2015)	ETZ	Android	(i) To access the pain in dementia patients.	Health and Safety Monitoring	No	1000+ downloads (4.2)

Brain Training Journey (2015)	Jarrod Kanizay	iOS	(i) Endless game levels to perform simple exercises. (ii) Memory oriented game.	Cognitive Training (Memory)	No	No data given

Care Tracker (2015)	Stella Care ApS	Android & iOS	(i) To help carers in tracking dementia patients	Navigation	Yes	1000+ downloads (4.7)

Dementia Care Matters (2015)	Appsme Ltd	iOS	(i) Organizational project. (ii) Push notification enabled. (iii) Integrated Facebook and Twitter feeds.	Daily Living Activities (Scheduling)	No	No data given

Dementia Test-Risk Calculator of dementia (2015)	Pears Health Cyber s.r.o.	iOS	(i) Dementia risk test. (ii) Disease information. (iii) Option to send test results via email. (iv) Next test date reminder. (v) Tips and tricks to reduce the risk of dementia.	Daily Living Activities, Dementia Screening	No	No data given

Memory Box (2015)	Swedish Care International AB	Android & iOS	(i) Provide daily interactions between caregivers, family and PwD.	Leisure and Socialization	Yes	1000+ downloads (4.9)

Dementia/Digital Diary/Clock (2015)	Fashmel	Android	(i) Provide digital/analog clock display. (ii) Live and remotely configured calendar.	Daily Living Activities	Yes	1000+ downloads (4.4)

Dementia Clock (2015)	Wearing the Green	Android	(i) Display interactive clock for PwD.	Daily Living Activities	No	1000+ downloads (4.5)

Care4Dementia (2015)	University of New South Wales	iOS	(i) Provide information and support for carers in their role of caring for PwD.	Carer Support	No	1000+ downloads (4.2)

GreyMatters: Beyond Dementia (2015)	GreyMatters Care LLC	iOS	(i) Creates personalized life storybook. (ii) Record and share memories. (iii) Listen to music and play games. (iv) Collaborate with family. (v) Record and share new memories.	Leisure and Socialization (Reminiscence Therapy)	Yes	18 reviews (4.5)

Living and Dying Well With Dementia (2015)	Footsqueek	iOS	(i) Developed by experts in the field including from the Alzheimer's Society UK, Gold Standards Framework and the End of Life Partnership (ii) Provide support to carers and identify key issues in Dementia and End of Life Care	Carer Support	No	No data given

Consult Geri: Dementia (2015)	New York University	iOS	(i) Joint project with Hartford Institute for Geriatric Nursing (HIGN). (ii) Guide for caregivers of PwD.	Carer Support	No	No data given

Care and Connect: Dementia Friendly Places (2015)	University of Newcastle upon Tyne	iOS	(i) Developed as part of a research project at Newcastle University. (ii) Find dementia friendly places and leave reviews.	Leisure and Socialization	Yes	No data given

Sea Hero Quest (2016)	GLITTERS LLD	Android & iOS	(i) A game for tracking the navigation data.	Health and Safety Monitoring	Yes	2.8k reviews (4.6)

Jigsaw Puzzle: Spider (2016)	CoCoPaPa Soft	Android	(i) 50 free puzzle images of spider to solve. (ii) Allow to zoom-in, zoom-out, move-in screen.	Cognitive Training (Problem Solving)	No	1000+ downloads (4.8)

Dementia Risk Tool (2016)	RISE SISC	Android & iOS	(i) Developed with Ageing Research Center at Karolinska Institute. (ii) To estimate the risks of developing dementia in the future.	Dementia Screening	Yes	100+ downloads (4.8)

Dementia guide for carers and care providers (2016)	Text Matters Ltd	iOS	(i) Centre for Information Design Research, University of Reading Project. (ii) To understand dementia. (iii) Day-to-day living. (iv) To support caregivers. (v) To provide Legal and Money Information. (vi) To provide symptoms and behaviours. (vii) To give knowledge about medical terms.	Carer Support	Yes	No data given

MindMate- Health ageing (2016)	MindMate Ltd	iOS	(i) Brain work out. (ii) Nutrition. (iii) Exercise. (iv) Reminder. (v) Games based on: Problem-solving. Speed. Memory. Attention.	Cognitive Training and Daily Living Activities	Yes	425 reviews (4.7)

BrainScore: Connect Dots (2016)	DAEMYEONG KONG	iOS	(i) Memory training app with checking attention skills of a dementia patient.	Cognitive Training (Attention, Memory)	No	No data given

A walk through Dementia (2016)	Alzheimer's Research UK	Android & iOS	(i) Provide virtual reality-based experience of dementia to understand PwD better.	Health & Safety Monitoring, Daily Living Activities	No	5000+ downloads (4.9)

Folstein Test (2016)	Openmylab	Android	(i) MMSE or Folstein test to measure cognitive impairment. (ii) Data saving options. (iii) Audio record observations.	Dementia Screening	Yes	1000+ downloads (4.6)

My House of Memories (2016)	National Museums & Galleries on Merseyside	Android	(i) Explore objects from the 1920s to 1980s. (ii) Share Memories, Experience music (iii) Activities to do together by carer and dementia patient.	Leisure and Socialization (Relaxation, Reminiscence Therapy)	Yes	1000+ downloads (4.2)

Dementia Caregiver Application v2 (2016)	Melvyn Zhang Weibin	iOS	(i) Joint project with the University of Newcastle School of Nursing. (ii) Provide caregiving tips and current stress index of carers.	Carer Support	No	No data given

myPlayLife (2017)	myPlayLife Ltd	iOS	(i) Piloted in 16 home cares. (ii) Create a private network of family members. (iii) Share photos, videos and audios.	Leisure and Socialization (Reminiscence Therapy)	Yes	No data given

Brain Test (2017)	Brain Test Inc.	iOS	(i) To detect cognitive changes associated with dementia and MCI.	Dementia Screening	Yes	56 reviews (4.6)

CogniCare-Enabling personalized dementia care (2017)	Cognihealth	Android	(i) Monitor symptoms, record care challenges. (ii) Enable to communicate better with the care worker.	Health & Safety Monitoring	Yes	1000+ downloads (5)

REMEMBER ME (2017)	Cuzzrek	Android	(i) Helps PwD to remember the name of family members.	Cognitive Training (Remember)	No	1000+ downloads (4.9)

Alzheimer's Speed of Processing Game-ASPEN (2017)	Tiny Happy Steps	Android	(i) Cognitive training app.	Cognitive Training (Logical Thinking, Memory)	No	1000+ downloads (4.3)

Cognitive Rehab in Dementia (2017)	NHS Education for Scotland	Android & iOS	(i) NHS Education for Scotland (NES) Project. (ii) Provide two sections: *Learn Section*: Provides educational information in cognition, cognitive impairment and process of cognitive rehabilitation. *Rehab Section*: Guide users through the process of cognitive rehabilitation for a person with early-stage dementia.	Carer Support	No	100+ downloads

eSLUMS (2017)	Chewy Logic, LLD	iOS	(i) Saint Louis University School of Medicine. Project. (ii) East test administration of iPad/iPhone. (iii) Automatic Scoring. (iv) Quickly see trends in test scores. (v) A dark and light theme for easy rating. (vi) Outline/Offline functions. (vii) Viewing results on-the-go with an iPhone. (viii) View and manage patient history.	Dementia Screening	No	22 reviews (4.2)

SymptomGuide™ Dementia (2018)	DGI Clinical	iOS	(i) DGI Clinical Project. (ii) Help caregivers in learning about dementia.	Carer Support	No	No data given

Dementia Respond (2018)	Academy for Professional Excellence	Android	(i) Academy for Professional Excellence's San Diego project. (ii) To provide carers to get information about dementia disease.	Carer Support	No	10+ downloads (5)

Your Care Card (2018)	The Memory Kit, LLC	iOS	(i) Connect all the care team members (ii) Designed to help carer better understand a person with dementia	Carer Support	Yes	No data given

## Data Availability

Please contact authors for data requests.
